# An improved method for measuring metaldehyde in surface water using liquid chromatography tandem mass spectrometry

**DOI:** 10.1016/j.mex.2016.03.004

**Published:** 2016-03-10

**Authors:** Melanie Schumacher, Glenn Castle, Anthony Gravell, Graham A. Mills, Gary R. Fones

**Affiliations:** a Natural Resources Wales, Llanelli Laboratory, 19 Penyfai Lane, Llanelli SA15 4EL, UK; b School of Earth and Environmental Sciences, University of Portsmouth, Burnaby Building, Burnaby Road, Portsmouth PO1 3QL, UK; c School of Pharmacy and Biomedical Sciences, University of Portsmouth, St Michael’s Building, White Swan Road, Portsmouth PO1 2DT, UK

**Keywords:** An improved method for measuring metaldehyde in surface water using liquid chromatography tandem mass spectrometry, Metaldehyde, Surface water, Liquid chromatography–tandem mass spectrometry, On-line enrichment, Methylamine, Molluscicide

## Abstract

The molluscicide metaldehyde (2,4,6,8-tetramethyl-1,3,5,7-tetraoxocanemetacetaldehyde) is an emerging pollutant. It is frequently detected in surface waters, often above the European Community Drinking Water Directive limit of 0.1 μg/L for a single pesticide. Gas chromatography mass spectrometry (GC–MS) can be used to determine metaldehyde in environmental waters, but this method requires time consuming extraction techniques prior to instrumental analysis. Use of liquid chromatography-tandem mass spectrometry (LC–MS/MS) can overcome this problem. We describe a novel LC–MS/MS method, using a methylamine mobile phase additive, coupled with on-line sample enrichment that allows for the rapid and sensitive measurement of metaldehyde in surface water. Only the methylamine adduct of metaldehyde was formed with other unwanted alkali metal adducts and dimers being suppressed. As considerably less collision energy is required to fragment the methylamine adduct, a five-fold improvement in method sensitivity, compared to a previous method using an ammonium acetate buffer mobile phase was achieved. This new approach offers:

• A validated method that meets regulatory requirements for the determination of metaldehyde in surface water.
• Improved reliability of quantification over existing LC–MS/MS methods by using stable precursor ions for multiple reaction monitoring.
• Low limits of quantification for tap water (4 ng/L) and river water (20 ng/L) using only 800 μL of sample; recoveries > 97%.

## Graphical abstract

**Figure:**
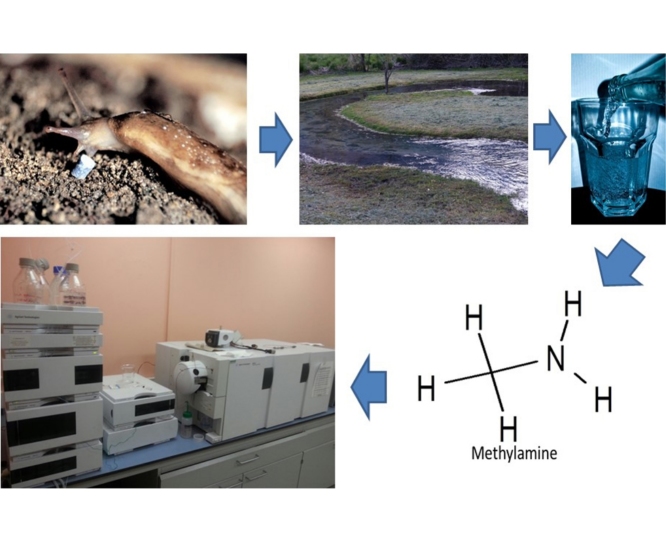

## Method details

### Reagents and standards

Acetonitrile and methanol of LC–MS grade purity were from VWR International Ltd. (Lutterworth, UK). Deuterated metaldehyde-d_16_ (>99 atom% deuterium) was from QMX Laboratories Ltd. (Thaxted, UK). Metaldehyde (99%) and methylamine (2 M) were from Sigma-Aldrich Ltd. (Gillingham, UK). Ultrapure water (18 MΩ ∙ cm) was used throughout and was produced from an Elga Purelab Prima water system (High Wycombe, UK).

Glassware is cleaned using a 10% Decon-90 solution (Decon Laboratories Ltd., Hove, UK), then rinsed with tap water, ultrapure water and finally methanol. Metaldehyde stock solution is prepared by dissolving 25 mg metaldehyde powder in 25 mL methanol to give a concentration of 1 g/L. The solution is kept in the dark at room temperature. Subsequent dilutions in methanol are undertaken to produce a final concentration of 50 μg/L, this solution is used to produce the aqueous calibration standards.

An internal standard stock solution (deuterated metaldehyde-d_16_) is prepared by dissolving 10 mg of the powder in 10 mL methanol to give a concentration of 1 g/L. Subsequent dilutions in methanol are undertaken to produce a final concentration of 50 μg/L. Each sample and calibration standard prepared in ultrapure water is spiked with internal standard to give a concentration of 1 μg/L.

A 2.5 mM methylamine + 0.05% acetic acid solution is prepared in a fume hood by adding 250 μL of acetic acid and 625 μL of 2 M methylamine to 500 mL of ultrapure water. This solution is freshly prepared every 3 days.

### LC–MS/MS instrumentation

All measurements are performed using an Agilent 1260 Infinity LC system comprising a 6460 triple quadrupole (Part No. G6460A) equipped with a jet stream electrospray ionisation source (Part No. G1958-65138), vacuum degasser (Part No. G1379B), binary pump (Part No. G1312B) and thermostated column compartment (Part No. G1316A). The LC system, mass spectrometer and data analysis are controlled using Agilent Mass Hunter software version B.05.01. (Agilent Technologies, Santa Clara, USA).

The analytical column is an Atlantis T3 (C_18_), 2.1 mm × 50 mm, 3 μm particle size (Part No. 186003717), with an Atlantis T3, 2.1 mm × 10 mm used as guard column, (Part No. 186003756), Waters, Elstree, UK). The mobile phase consists of an aqueous 2.5 mM methylamine + 0.05% acetic acid solution (solvent A) and acetonitrile (solvent B) at a flow rate of 0.3 mL/min and is used in the gradient elution mode. Mass spectrometer source conditions and solvent elution conditions are shown in Table 1, Table 2 respectively. Total run time is 8 min.

### On-line sample enrichment

**Fig. 1:**
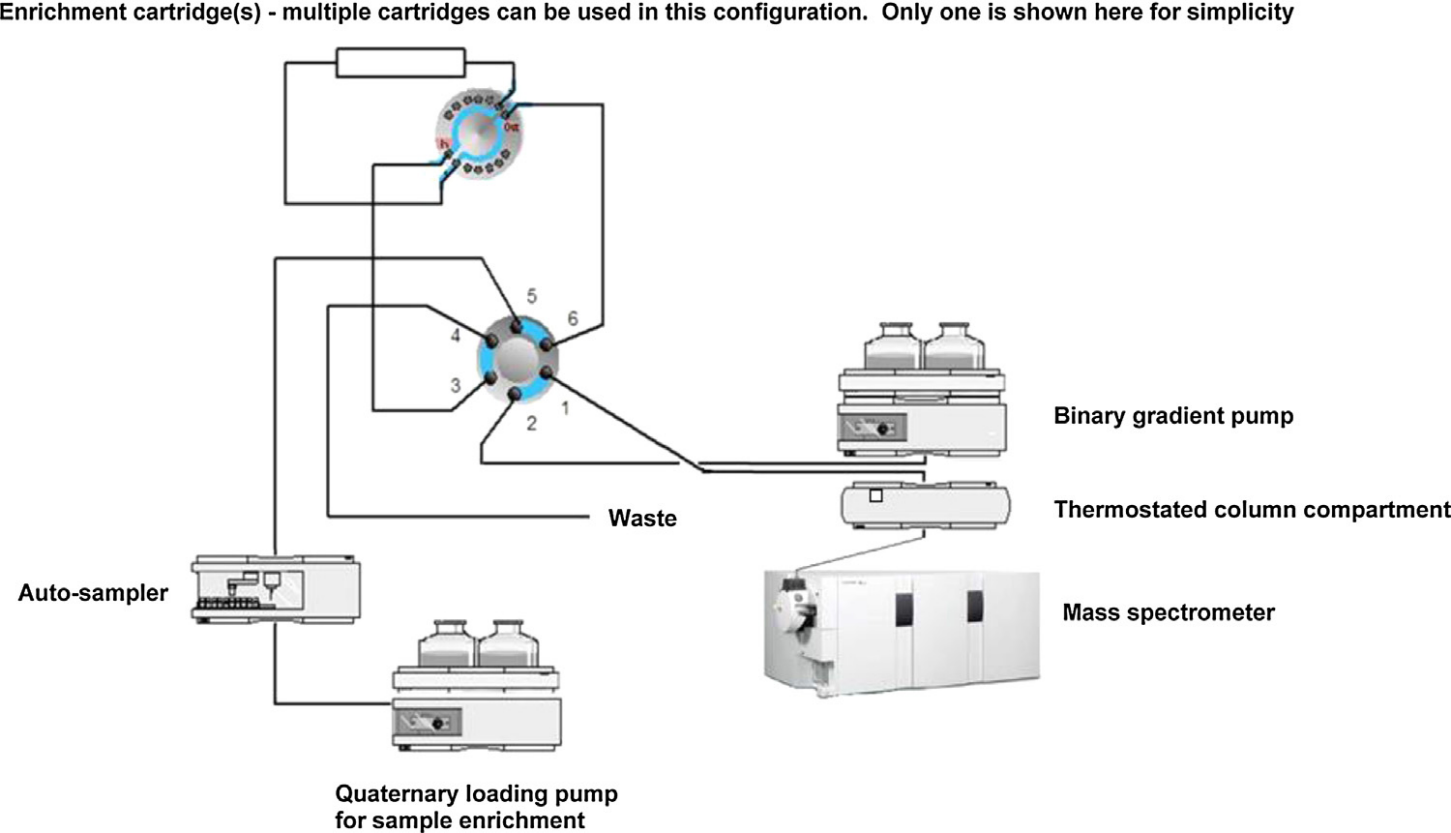
Schematic of the on-line enrichment system shown in sample load position.

The sample is introduced via an on-line enrichment system comprising a standard Agilent 1260 Infinity quaternary pump, Agilent 1260 auto-sampler and a programmable Agilent 1200 Infinity 12 port/6-position selection valve (Part No. G1159A). A second programmable Agilent 1200 Infinity 2-position/6-port valve (Part No. G1158A) is used to select between loading onto a re-useable solid-phase extraction (SPE) cartridge or elution onto the analytical column. The entire on-line enrichment system is fully integrated and controlled by the Agilent Mass Hunter software. 800 μL aliquot of sample is introduced into the loading line via the auto-sampler and pumped using the quaternary pump onto a Waters re-usable Oasis HLB on-line SPE cartridge (2.1 mm × 10 mm) (Part No. 186005786). Following sample enrichment the SPE cartridge is eluted (back-flushed) by the binary pump gradient onto the analytical column for separation and detection of metaldehyde by the mass spectrometer. The on-line programme for the conditioning, loading and elution of SPE cartridge is shown in Table 3. The on-line system is shown schematically in Fig. 1.

### Sample preparation

Dilute stock metaldehyde solution (50 μg/L) in ultrapure water to obtain working calibration standard solutions of concentration 0, 10, 50, 100, 300, 500, 750 and 1000 ng/L

Attach an Oasis HLB cartridge to the 12 port/6-position selection valve.

Add 1 mL of sample and working calibration standard solutions into labelled silanized auto-sampler vials. Add 20 μL of internal standard solution (50 μg/L) to each vial and mix. Transfer to auto-sampler tray.

Load method which contains the entire LC conditions and switching valve programme, as shown in Table 3, via the Mass Hunter data acquisition software.

Allow system to stabilise for 30 min prior to commencing analysis. Ensure that the on-line enrichment and HPLC system is leak free.

Analyse samples and calibration standards solutions and prepare a calibration Table via the Mass Hunter data analysis software.

### Data acquisition

Data acquisition was performed in multiple reaction monitoring mode (MRM) between 1.8–3.0 min using the conditions shown in Table 4.

### Quantification

The ion transitions *m*/*z* = 208.2 to *m*/*z* = 76.1 and *m*/*z* = 208.2 to *m*/*z* = 176 are used for quantification and qualification of metaldehyde respectively. The ion transition for the internal standard is *m*/*z* = 224.3 to *m*/*z* = 80.2. The calibration curve for metaldehyde is obtained by injecting standards at concentrations of 0, 10, 50, 100, 300, 500, 750, 1000 ng/L. These are made up from stock solutions as described above and spiked with internal standard (1 μg/L) prior to analysis. Linear regression is applied to the internally standardized calibration plot with a weighting factor of 1/*x* which results in a better fit of the values for the lower concentration standards. The correlation coefficient (*R*^2^) for the eight point calibration is typically > 0.999.

### Validation

Validation was undertaken using the Water Research Centre NS30 protocol. This is accepted as best practice within the water industry in the UK for the derivation of the accuracy requirements of an analytical system when monitoring to a particular water quality standard [1]. NS30 requires a series of tests to be carried out to assess the precision of analysis across the analytical range, over a period of two weeks or longer. It is also consistent with the specifications made within ISO/TS 13530:2009. Limits of quantification (LoQ) of 4 and 20 ng/L were obtained using tap water and river water matrices respectively. Recoveries of metaldehyde from both matrices were > 97%. A summary of the validation data is shown in Table 5.

The method was further validated by participation in Aquacheck, an analytical proficiency testing scheme provided by LGC and accredited by the UK Accreditation Service (UKAS). The result of 68.3 ng/L obtained by the new method showed good agreement with the assigned value for metaldehyde of 60.1 ng/L, well within the Z-score threshold of ±2 to pass the test.

## Additional information

Metaldehyde is an emerging pollutant in environmental waters. It is used to control slugs and snails in a wide range of agricultural, horticultural and domestic crops and is the most widely used molluscicide in the UK. Metaldehyde is very stable, not readily biodegradable and is frequently found in the aquatic environment at concentrations far exceeding the European Community Drinking Water Directive limit of 0.1 μg/L [2]. During 2012, fourteen water companies in England recorded a total of 232 regulatory exceedances for metaldehyde. Such exceedances have led to water companies being required to effectively monitor the sources and fluxes of metaldehyde in the aquatic environment under their management [3].

Typically GC–MS, using single or triple quadrupole instruments, is used to determine metaldehyde in water at concentrations above or below the Drinking Water Directive limit [4]. GC–MS methods require metaldehyde to be extracted from the water using time consuming off-line techniques such as liquid–liquid or solid-phase extraction prior to analysis [4], [5].

Using an Agilent LC–MS/MS we successfully modified a previous instrumental method used for the analysis of metaldehyde in water. Initially, the choice of the mobile phase buffer on the ionisation and fragmentation processes of metaldehyde was evaluated. The objective was to improve method sensitivity for measuring metaldehyde by overcoming the unfavourable conditions obtained by the formation of multiple adduct ions when using ammonium acetate as the conventional mobile phase buffer. This modified method uses an alkyl-ammonium buffer (methylamine) as a mobile phase additive. Using this buffer, the methylamine-adducted metaldehyde was observed as the only major molecular ion, while the formation of other adduct ions especially ([M + H]^+^, [M + Na]^+^, [M + NH_4_]^+^, [M + K]^+^) and dimers were highly suppressed. Also, product ion spectra with a single major fragment ion were not seen, unlike that observed with ammonium acetate buffer.

**Fig. 2:**
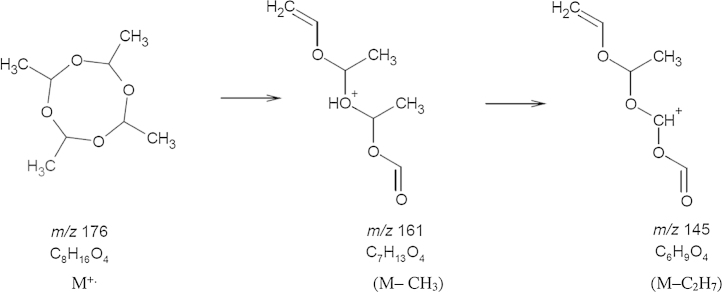
Proposed fragmentation pathway for the ion observed at *m*/*z* = 145 obtained from ACD/MS Fragmenter software.

The affinity of alkyl-ammonium buffers and their basicity towards compounds are believed to be factors that influence the formation and abundance of molecular and fragment ions, respectively [6], [7], [8]. Methylamine appears to have a strong affinity towards metaldehyde and the binding energy between the two is greater than that of other adducts, effectively suppressing their formation. The methylamine adduct ion (*m*/*z* = 208.2) of metaldehyde (M) has the following formula [M + CH_3_NH_2_]^+^ and is the primary adduct formed. This undergoes fragmentation in the collision cell of the mass spectrometer to form the methylamine adduct of ethanal observed at *m*/*z* = 76.2 in a product ion scan. Other ions observed in the product ion scan include *m*/*z* = 176.1, which is probably due to the removal of methylamine moiety to yield the molecular ion of metaldehyde, and *m*/*z* = 145 which is the loss of C_2_H_7_, from the molecular ion to form C_6_H_9_O_4_^+^. The hypothesis for the formation of the ion at *m*/*z* = 145 is supported by the use of ACD/MS Fragmenter software (Toronto, Canada) (see Fig. 2).

Overall, better precision (<7.0% RSD at the Drinking Water Directive limit of 0.1 μg/L) and a five-fold improvement in method sensitivity were obtained for metaldehyde when using the methylamine buffer compared with the previously used ammonium acetate buffer. Compared to off-line liquid–liquid or solid-phase extraction the new method offers significant benefits in terms of lower sample volumes, speed and cost of analysis. The methylamine buffer effectively eliminates the formation of problematic alkali metal adducts by forming the one methylamine adduct and can be used for other compounds where unwanted alkali metal adducts are formed. The new method has now been in routine use for over 6 months analysing several hundred surface water samples for regulatory reporting purposes. This easily to implement method, requiring only a simple modification to the mobile phase, should prove attractive to analysts based in laboratories of water companies and environmental regulators.

## Figures and Tables

**Table 1:** Mass spectrometer source conditions.

Gas Temp (°C)	250
Gas Flow (L/min)	5
Nebuliser pressure (psi)	60
Sheath gas heater (°C)	300
Sheath gas flow (L/min)	11
Capillary voltage (V)	3000
Nozzle voltage (V)	1000

**Table 2:** Solvent elution timetable.

Time (min)	Solvent B (%)
0	30
3	67.5
3.5	100
4.5	100
5.0	30

**Table 3:** On-line SPE conditions.

Mobile phase	A: Water (2.5 mM methylamine + 0.05% acetic acid)	
	B: Acetonitrile	
Temperature	Ambient	
Mobile phase (quaternary/loading pump)	A: Water	
	B: Acetonitrile	
Quaternary pump sample loading flow (mL/min)	1.0	
Sample loading flow (mL/min)	1.0	
Injection volume (μL)	800	
Gradient programme:	Time (min)	(% solvent B)
	0.0	0
	0.5	100
	5.0	100
	5.5	0
	7.7	0
	8.0	0
Injector Programme:	Command	
	DRAW: defined amount from sample from vial (800 μL)	speed 500 μL/min
	VALVE: main-pass	
	WAIT: 3.5 min	
	REMOTE: start pulse	
	WAIT: 2.0 min	
	EJECT: defined amount into seat	speed 900 μL/min
2-position/6-port valve set-point timetable	Time (min)	Position
	0.0	2
	0.1	1 (elution)
	2.0	2 (conditioning)

**Table 4:** LC–MS/MS acquisition conditions.^a^

Compound	Precursor mass (*m*/*z*)	MS resolution	Product mass (*m*/*z*)	Dwell time (ms)	Fragmentor voltage (V)	Collision energy (eV)	Cell acceleration voltage (V)
Metaldehyde-d_16_	224.3	Unit	80.2	250	135	3	7
Metaldehyde (Quantitative)	208.2	Unit	76.1	250	135	3	7
Metaldehyde (Qualitative)	208.2	Unit	176.1	250	135	3	7

a The mass spectrometer was operated in positive electrospray and multiple reaction monitoring (MRM) mode.

**Table 5:** Summary of validation data for the on-line LC–MS/MS method.

Matrix	Level	Spiked conc. (ng/L)	Measured conc. (ng/L)	Batches	DoF	LoD rounded (ng/L)	LoQ	% RSD	% Bias	% Rec	% UoM
Tap water	Unspiked	–	–	11	11	2.0	4.0	–	–	–	25.1
	Low spike	100.0	99.0	11	13			6.8	−1.0	97.6	
	High spike	750.0	743.0	11	16			4.5	−1.0	98.9	

River water	Unspiked	–	–	11	14	9.0	20.0	–	–	–	27.1
	Low spike	108.0	108.0	11	20			7.8	0.1	100.0	
	High spike	758.0	748.0	11	15			4.5	−1.4	98.6	

The tap water and river water used for validation experiments contained measurable field incurred residues of metaldehyde which were taken into account when calculating the LoD and LoQ. Key: DoF = degrees of freedom, LoD = limit of detection, LoQ = limit of quantification, RSD = relative standard deviation, Rec = recovery, UoM = uncertainty of measurement.
